# Androgen Deprivation Therapy Combined With Particle Therapy for Prostate Cancer: A Systematic Review

**DOI:** 10.3389/fonc.2021.695647

**Published:** 2021-06-23

**Authors:** Stine Elleberg Petersen, Morten Høyer

**Affiliations:** Danish Centre for Particle Therapy, Aarhus University Hospital, Aarhus, Denmark

**Keywords:** prostate cancer, androgen deprivation therapy, biochemical disease-free survival, overall survival, acute morbidity, late morbidity, proton therapy, carbon ion therapy

## Abstract

**Purpose:**

There is high-level evidence for addition of androgen deprivation therapy to photon-based radiotherapy of the prostate in intermediate- and high-risk prostate cancer. Little is known about the value of ADT in particle therapy of prostate cancer. We are conducting a systematic review on biochemical disease-free survival, overall survival, and morbidity after combined particle therapy and ADT for prostate cancer.

**Methods:**

A thorough search in PubMed, Embase, Scopus, and Web of Science databases were conducted, searching for relevant studies. Clinical studies on prostate cancer and the treatment combination of particle therapy and androgen deprivation therapy were included. The review was conducted according to the Preferred Reporting Items for Systematic Reviews and Meta-Analyses (PRISMA) guidelines and registered on PROSPERO (CRD42021230801).

**Results:**

A total of 298 papers were identified. Fifteen papers reporting on 7,202 patients after proton or carbon-ion therapy for localized prostate cancer where a fraction or all patients received ADT were selected for analysis. Three thousand five hundred and nineteen (49%) of the patients had received combined ADT and particle therapy. Primarily high-risk (87%), to a lesser extent intermediate-risk (34%) and low-risk patients (12%) received ADT. There were no comparative studies on the effect of ADT in patients treated with particles and no studies identified ADT as an independent prognostic factor related to survival outcomes.

**Conclusions:**

The review found no evidence to support that the effects on biochemical disease-free survival and morbidity of combining ADT to particle therapy differs from the ADT effects in conventional photon based radiotherapy. The available data on the topic is limited.

## Introduction

Prostate cancer (PC) is the second most common cancer among men worldwide, however with a relatively high survival rate (1). The efficacy of radiotherapy (RT) of PC was demonstrated in the randomized Scandinavian Prostatic Cancer Group 7 (SPCG-7) trial where intermediate- and high-risk patients receiving 70 Gy to the prostate had superior survival outcomes compared to patients who did not receive RT (2). In the SPCG-7 study, patients in both randomization arms received 3 months of neoadjuvant androgen deprivation therapy (ADT) and life-long antiandrogen. The European Organization for Research and Treatment of Cancer (EORTC) 62863 Study randomized high-risk patients who all received pelvic RT to either long-term (3 years) ADT or no ADT showed that concomitant/adjuvant ADT improved 10-years overall survival (OS) from 40% (95% CI 32–48%) to 58% (95% CI 49–66%) in high-risk PC patients (3). Short-term ADT (6 months), however, provides inferior OS as compared with 3-years of ADT as demonstrated in the EORTC 22961 Study (4). In intermediate-risk PC patient, the EORTC 22991 Study showed that combined RT plus short-term ADT (6 months) increased biochemical disease-free survival (bDFS) (5).

In most studies on the efficacy of concomitant/adjuvant ADT, the patients received conventional RT doses (60–70 Gy) that are now considered inadequate. Dose-escalation studies randomizing between 70 Gy or 74–78 Gy with long-term follow-up have shown improved bDFS with high dose (6–10) and a very recent study on simultaneous integrated boost with up to 95 Gy to focal intraprostatic lesions further improved bDFS (11). Studies of escalated proton boost to the prostate from the Massachusetts General Hospital revealed improved bDFS in patients receiving the escalated dose (12). The escalated radiation dose to the prostate may to some extent neutralize the effect of concomitant/adjuvant ADT, but guidelines still recommend the same concomitant/adjuvant ADT combined with high-dose radiation therapy (13).

Particle therapy is used to optimize the therapeutic ration in treatment of PC, improving disease control and minimizing treatment related morbidities. However, little is known about the interaction between particle therapy and ADT and the outcomes regarding bDFS, overall survival, and morbidity for PC patients. We conducted a review on the efficacy of the combination of particle therapy and ADT and the morbidity following this combined treatment.

## Methods

### Literature Search Strategy and Data Sources

The systematic review process was carried out following the Preferred Reporting Items for Systematic Reviews and Meta-Analyses (PRISMA) guidelines (14, 15); the protocol is registered in the PROSPERO database (registration number CRD42021230801). On January 11, 2021 a thorough database literature search was performed on PubMed, Embase, Web of Science, and Scopus to identify relevant publications. The following keywords were used: prostate neoplasms, prostate cancer, prostate carcinoma, prostate tumor, proton, proton therapy, carbon ion therapy, androgen antagonists, androgen depravation therapy, and antiandrogen.

### Study Selection

To identify relevant studies, the following inclusion criteria were used: original study, clinical study, ADT combined with particle therapy in primary therapy for prostate cancer, and reporting of treatment outcomes (see below). The following exclusion criteria were used: language other than English, Danish, Swedish, or Norwegian, reviews, meta-analyses, guidelines, editorials, comments, case reports, letters to and communications without original data, and conference abstracts. If publications of identical patient cohorts were found, the most complete study was chosen.

### Interventions

The interventions were particle therapy and ADT to patients with PC in a curative setting. Neoadjuvant, adjuvant, and the combination of neoadjuvant plus adjuvant ADT were considered.

### Outcomes

Biochemical disease-free survival, OS, acute and late morbidity, and patient reported outcome measures (PROs) were outcomes of interest. Biochemical disease-free survival was defined by the Phoenix criteria (16) and OS was defined in the individual papers. Morbidity and PROs were any morbidity described in the papers regarding grade and timing (acute or late).

### Data Extraction and Quality Assessment

For initial inclusion in the study, the two authors (MH and SP) independently screened titles and abstract, in case of disagreement, consensus was reached. Full text screening and data extraction was performed by one of the authors (SP) and checked by the other author (MH). Endnote (17) was used to manage study selection and Covidence (18) was used in the process of inclusion and exclusion of papers.

## Results

### Study Selection and Patients’ Characteristics

**Figure 1:**
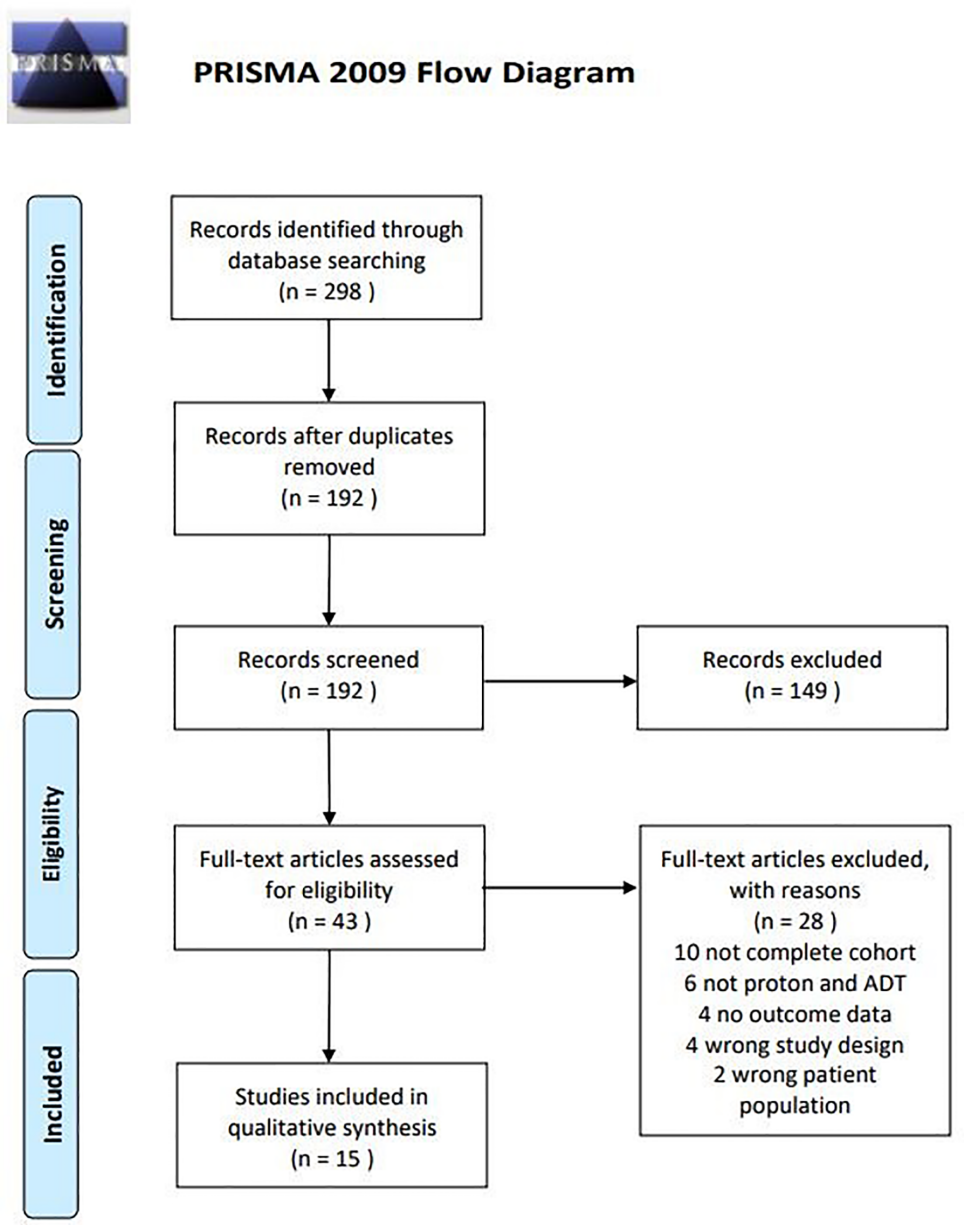
Flow chart of inclusion and exclusion procedure.

The literature search revealed a total of 298 papers. After removal of duplicates, 192 papers were eligible for review. Titles and abstracts were screened for eligibility based on the intervention and outcomes, 40 papers were eligible for full-text screening. Of the 40 papers, 15 papers met the selection criteria for analysis (**Figure 1**). Of the 15 studies, nine had a prospective design, four had a retrospective design, and one study was a match-pair analysis. The number of included patients ranged from 58 to 2,021 and follow-up time varied from 1 year to 7 years. Seven studies reported on bDFS and OS. All studies reported on morbidity in terms of one or more of the following: toxicity scoring, PROs, and QoL.

The 15 papers reported on a total of 7,202 patients. Of these, 1,411 (20%) had low-risk, 2,837 (41%) intermediate-risk, and 2,639 (39%) high-risk disease. Patients received proton only in seven studies, carbon ions in four studies, combined protons and photons in three of the studies, and protons or carbon ions in one study. Total dose and dose per fraction fractionation ranged 51.6–82 Gy (RBE) and 1.8–5 Gy (RBE), respectively (**Table 1**). The majority of patients received treatment on prostate only, in two of the studies 34 (3%) and 5 (6.6%) of the patients received prophylactic pelvic irradiation (**Table 1**). Across all included studies, 3,519 (49%) patients received ADT. In studies reporting D’Amico risk-group, 12% (range between studies: 0–22%) of the low-risk, 34% (0–67%) of the intermediate-risk, and 87% (66–100%) of the high-risk patients received ADT (**Table 2**). The median duration of ADT was only reported in six studies, ranging from 6 to 30.2 months.

**Table 1:** Biochemical disease-free survival and overall survival.

Paper	N	Study design	Modality	Risk groups No. (%)	FU (years)	Dose (Gy)	PO/WP	ADT No. (%)	bDFS (% at 5 years)	OS (% at 5 years)
Takagi et al. (19)	2,021	Retrospective observational	Proton	LR 335 (17)	7	74–78 Gy*	2,021/0	LR 63 (19)	90**	96**
								IR 373 (42)		
				IR 894 (44)				HR 709 (90)		
				HR 792 (39)						
Bryant et al. (20)	1,327	Prospective	Proton	LR 547 (41)	5.5	75–82 Gy*	1,327/34	LR 37 (7)	LR 99	LR 98
				IR 551 (42)				IR 56 (10)	IR 94	IR 97
				HR 229 (17)				HR 151 (66)	HR 74	HR 95
Goenka et al. (21)	81	Prospective	Proton	LR 32 (40)	0.5	79.2 Gy^#^	81/5	LR 0 (0)	NA	NA
				IR 32 (40)				IR 6 (19)		
				HR 17 (21)				HR 16 (94)		
Habl et al. (22)	91	Prospective randomized	Proton/carbon ion	LR 21 (23)	1.9	66 Gy^¤^	92/0	21 (22.8) ^Π^	NA	NA
				IR 54 (59)						
				HR 16 (17)						
Kasuya et al. (23)	324	Prospective	Carbon ion	HR 324	9	63–66 Gy^¤^	324/0	HR 324 (100)	88	93
Nomiya et al. (24)	46	Phase I/II clinical trial	Carbon ion	LR 12 (26)	2.7	51.6 Gy	46/0	45 (98)^Π^	NA	NA
				IR 9 (20)						
				HR 25 (54)						
Johansson et al. (25)	278	Prospective	Proton+photon	LR 63 (24)	4.8	50Gy + 20Gy*^£^	278/0	LR 14 (22)	LR 100	LR 90
				IR 95 (36)				IR 43 (45)	IR 95	IR 90
				HR 107 (40)				HR 81 (76)	HR 74	HR 87
Grewal et al. (26)	184	Prospective	Proton	LR 18 (10)	4.2	78 Gy^£^	184/0	LR 0 (0)	93.5**^$^	95.8**^$^
				IR 166 (90)				IR 47 (28)		
Zhang et al. (27)	64	Prospective	Carbon ion	LR 3 (5)	1.6	59.2–66 Gy^β^	64/0	LR 0 (0)	NA	NA
				IR 24 (38)				IR 16 (67)		
				HR 37 (16)				HR 19 (73)		
Yu et al. (28)	314	Retrospective	Proton *vs.* IMRT	Na	1	NA	NA	65 (21) ^Π^	NA	NA
Takakusagi et al. (29)	253	Prospective	Carbon ion	LR 8 (3)	2.9	51.6 Gy^¥^	253/0	244 (97) ^Π^	LR^α^ 98	NA
				IR 88 (35)					IR^α^ 88	
				HR 157 (62)					HR^α^ 88	
Mayahara et al. (30)	287	Prospective	Proton	LR 62 (22)		74 Gy*	287/0	204 (71) ^Π^	NA	NA
				IR 100 (35)						
				HR 125 (43)						
Matsukawa et al. (31)	583	Prospective	Proton	LR 72 (12)	4	70–78 Gy*	583/0	191 (33) ^Π^	NA	NA
				IR 268 (46)						
				HR 243 (42)						
Dutz A. et al. (32)	58	Match-pair analysis	Proton *vs.* IMRT	LR 2	1	74–78 Gy*	88/0	26 (30) ^Π^	NA	NA
				IR 45						
				HR 11						
Iwata et al. (33)	1,291	Retrospective	Proton	LR 215 (17)	5.8	63–80 Gy*^Ω^	1,291/0	LR 35 (16)	LR 97	LR 98
				IR 520 (43)				IR 244 (47)	IR 91	IR 97
				HR 556 (43)				HR 489 (88)	HR 83	HR 95

NA, not available; LR, low risk; IM, intermediate risk; HR, high risk; FU, follow-up; ADT, androgen deprivation therapy; PO, prostate only; WP, whole pelvic radiotherapy; *2 Gy/frx.; **for all patients; ^#^1.8 Gy/frx.; ^¤^3.3 Gy/frx.; ^$^at 4 years; ^£^5 Gy/frx; ^β^2.75–3.8 Gy/frx.; ^¥^4.3 Gy/frx.; ^α^at 3 years; ^Π^ADT use among all patients; ^Ω^3 Gy/frx.

**Table 2:** Use of ADT across studies (only eight studies reporting ADT included).

Risk groups	Number (%) in risk-groups	Number (%) receiving ADT
Low-risk	1,213 (22)	149 (12)
Intermediate-risk	2,282 (41)	785 (34)
High-risk	2,062 (37)	1,789 (87)
Total	5,557 (100)	2723

### Disease Free Biochemical Survival and Overall Survival

Of the seven publications reporting bDFS and OS, five stratified patients into low-, intermediate-, or high-risk groups according to D’Amico (34) and reported bDFS and OS for the three risk groups. The bDFS reported at 3–5 years ranged 87–100, 88–95, and 74–88% for the low-, intermediate-, and high-risk group, respectively. Overall survival rates at 5 years ranged from 90–98.4, 90–97, and 87–95.2% for the three risk groups (**Table 1**). The prognostic value of long-term (>24 months) ADT on bDFS was tested in a study on carbon-ion therapy for high-risk PC where long-term was not superior to short-term (<12 months) ADT (23). Androgen deprivation therapy was not tested as an independent prognostic factor in any of the studies included in the analysis.

### Morbidity Scores, Patient-Reported Outcomes, and Quality of Life

Seven of the publications reported acute toxicity and nine of the studies reported late morbidities according to the Common Terminology Criteria for Adverse Events (CTCAE) (19, 20, 22, 24, 26, 27, 29, 30, 32, 33). Incidences of grade ≥2 acute GI and GU morbidities ranged from 0–17 to 5–40%, respectively, and late grade ≥2 morbidities ranged from 0–14 to 0–32% for GI and GU morbidities, respectively (**Table 3**). In one study using the Radiation Therapy Oncology Group (RTOG) toxicity scale, the incidence of grade ≥2 late GI and GU morbidities after 5 years were 0 and 3–30% (25). With few exceptions, there were no significant changes in PROs and QoL endpoints from before to after RT in the seven publications reporting on these items. In one study sexual summary score declined significantly from baseline to 5 years follow-up (67 to 53) in patients not receiving ADT (20) and remained low and stable in patients receiving ADT. Another study revealed a significantly poorer urinary obstruction/irritation score at the end of treatment compared to baseline, but recovering afterwards (27), the same pattern was seen in one of the other publications using EPIC-26 for PROs (31).

**Table 3:** Acute and late morbidity and patient reported outcomes.

Paper	N	CTCAE GI (≥2)		CTCAE GU (≥2)		Other toxicity scales/PROs
		Acute	Late (5 years)	Acute	Late (5 years)	
Takagi et al. (19)	2,021	NA	4%	NA	2.2%	NA
Bryant et al. (20)	1,327	NA	0.6%*	NA	2.9%	EPIC-26: ns
						IPSS: ns
Goenka et al. (21)	81	NA	NA	NA	NA	EPIC-26: ns
						AUA: ns
Habl et al. (22)	91	7.7%** 4.4%***	NA	17.6%^¤^	NA	EORTC:
						QLQ-C30: ns
						PR25: ns
Kasuya et al. (23)	324	NA	NA	NA	NA	NA
Nomiya et al. (24)	46	0%	0%	4%	0%	NA
Johansson et al. (25)	278	NA	NA	NA	NA	RTOG gr2+ late (5 years):
						GU: 3–30%
						GI: 0%
Grewal et al. (26)	184	3.8%	13.6%^Ω^	12.5%	7.6%^Ω^	IPSS: ns
						IIEF-5: ns
						EPIC: ns
Zhang et al. (27)	64	0%	0%	10.9%	1.6%	EPIC-26: ns
						IPSS: ns
Yu et al. (28)	314	NA	NA	NA	NA	Tox 12 months (scale not specified):
						GU: 17.5–18.8%
						GI: 9.9–10.2%
Takakusagi et al. (29)	253	0%	1.2%^£^	4.7%	6.3%^£^	NA
Mayahara et al. (30)	287	0%	0%	40%	0%	NA
Matsukawa et al. (31)	583	NA	NA	NA	NA	EPIC-26: ns
Dutz A. et al. (32)	58	17%	9-14%^$^	27-44%	23-32%^$^	EORTC:
						QLQ-C30: ns
						PR25: ns
Iwata et al. (33)	1,291	NA	4.1%	NA	4.0%	NA

NA, not available; PRO, patient reported outcome; ns, not significant; GI, gastro-intestinal; GU, genitourinary; CTCAE, Common Terminology Criteria for Adverse Events; RTOG, The Radiation Therapy Oncology Group (35); EPIC-26, Expanded Prostate Cancer Index Composite (36); AUA, American Urological Association symptom score (37); IPSS, International Prostate Symptom Score (37); EORTC, European Organization for Research and Treatment of Cancer; QoL, questionnaire (EORTC QLQ-C30) (38); EORTC PR25 (39); IIEF, International Index of Erectile Function Questionnaire (40); *Grade ≥3; **proctitis; ***diarrhea; ^¤^cystitis; ^Ω^at 4 years; ^£^at 3 years; ^$^at 12 months.

## Discussion

The present review provides an overview of the available literature on combined particle therapy and ADT in therapy for PC. It included 15 reports on combined particle therapy and ADT. Across the studies, 48% of the patients received particle therapy in combination with ADT with a higher percentage (87%) of the patients in the high-risk group receiving ADT and with lower frequencies for intermediate- (34%) and low-risk groups (12%). The bDFS of 87–100, 88–95, and 74–88% and OS of for 90–98.4, 90–97, and 87–95.2% at 3–5 years for the low-, intermediate-, and high-risk group in the present review are comparable with outcomes of similar risk groups treated with photons in randomized clinical trials (3, 5, 41–43).

To a large extent, high-risk PC patients received ADT according to international guidelines that recommend long-term ADT (13). The use of ADT in intermediate-risk patients was less compliant to guidelines as only 34% of patients in this risk-group received ADT and the use of ADT in 7% of low-risk patients is not in accordance with guidelines. The reason for the non-compliance may be due to waiting lists, doctors’ or patients’ preferences.

Due to the quality of reporting, heterogeneity of study design, study cohorts, treatments, and dose-fractionation schedules, it was not possible to perform statistical metaanalysis of the effect of ADT on bDSF and OS. Review of the selected studies did not reveal evidence on the efficacy of combining ADT to particle therapy in PC. No randomized study addressed this issue and no study found that ADT was an independent prognostic factor related to survival outcomes. A study by Kasuya et al. on hypofractioned carbon ion therapy for high-risk PC tested the effect of length of ADT and it found no difference in bDSF in patients treated with short- and long-term ADT (23). However, in a previous publication from the same group on an overlapping patient population, long-term ADT resulted in improved bDFS compared to short-term ADT in very-high-risk patients (44). Due to the overlapping study populations, this study was not included in the present review.

The various rates of acute and late morbidity scores between the selected studies reflects the heterogeneous scoring schemes, administration by physicians or patients, patient groups, etc. The lack of detailed reporting of the studies did not allow assessment of the morbidity specifically related to ADT. Previous studies comparing morbidity of particle therapy to intensity modulated radiation therapy (IMRT) in PC patients revealed less GI morbidity in patients treated with particle therapy. The differences in morbidity in these studies were detected by the use of PROs (45, 46). However, in the present review we do not find data to support that ADT interacts with particles on development of radiation damage in a way that differs from photons. It is more likely that the observed toxicities are directly related to the radiation exposure of the organs at risk. However, in particle therapy, ADT may also contribute with specific hormonal related morbidity such as sexual dysfunction, hot flashes, fatigue, osteoporosis, metabolic syndrome, and potentially increased risk of cardiovascular disease (47).

Seven of the studies delivered particle therapy on moderate or ultra-hypofraction schedules. The heterogeneity of the data does not allow analysis of the effect of fractionation on the interaction between particle therapy and ADT. Follow-up in hypofractionated studies was short and does therefore not allow comparison of disease control and survival outcomes and there was no obvious difference in toxicity rates between studies with normo- and hypofraction. In randomized studies on photons, hypofractionation did not result in worse outcome compared to normofractioned RT (41, 42, 48, 49). It has been suggested that ADT is not needed in ultra-hypofractionation of PC. A study by King et al. on SBRT treating localized PC with 36.25 Gy in four to five fractions showed that there was no benefit of short-course ADT (50). In addition, ADT has never proven effective in high dose-rate (HDR) brachytherapy (51). There is so far no data suggesting a similar effect for particle therapy.

The current review has several limitations. The lack of comparative studies in particle therapy together with the heterogeneity of the included studies regarding design, particle therapy dose-fraction, toxicity scoring scales, and use of ADT represent major limitations of the present review. Furthermore, the quality on reporting on the use of ADT in specific risk groups, the duration of treatment, etc. are sparsely reported.

## Conclusions

Based on currently available literature, there is no evidence to support different use of ADT in particle therapy compared to standard of care in conventional photon RT of PC. Patients receiving particle therapy for PC should therefore receive ADT according to international guidelines, which implies use of short-term ADT for intermediate-risk and long-term for high-risk PC.

## Author Contributions

All authors listed have made a substantial, direct, and intellectual contribution to the work and approved it for publication.

## Conflict of Interest

The authors declare that the research was conducted in the absence of any commercial or financial relationships that could be construed as a potential conflict of interest.
